# Cutaneous Pain in Atopic Dermatitis: Mental Health Burden

**DOI:** 10.3390/jcm15082938

**Published:** 2026-04-13

**Authors:** Magdalena Kotewicz, Joanna Mazgaj, Andrzej K. Jaworek, Jacek C. Szepietowski

**Affiliations:** 1 Magdalena Kotewicz Private Practice, 50-203 Wrocław, Poland; mazgajmagda@gmail.com; 2 Dermatology Clinic, Görlitz Municipal Hospital, 02828 Görlitz, Germany; jmazgajj@gmail.com; 3 Department of Dermatology, Jagiellonian University, 31-503 Kraków, Poland; 4 Division of Dermatology, Venereology and Clinical Immunology, Faculty of Medicine, Wroclaw University of Science and Technology, 51-377 Wroclaw, Poland; 5 Department of Dermato-Venereology, 4th Military Hospital, 50-981 Wroclaw, Poland

**Keywords:** cutaneous pain, atopic dermatitis, depression, anxiety, quality of life, stigmatization

## Abstract

**Background:** Atopic dermatitis (AD) is a chronic inflammatory skin disease that is often associated with cutaneous pain. The objective of this study was to evaluate the impact of cutaneous pain on the prevalence of anxiety, depression, quality of life (QoL) and stigmatization in patients with AD. **Methods:** A cross-sectional study was conducted on a group of 113 adults with AD (61% females; mean age 34.48 ± 13.20 years). The severity of AD was evaluated using the Eczema Area and Severity Index (EASI). The intensity of cutaneous pain in the past week was measured using a Numerical Rating Scale (NRS), a Short Form McGill Pain Questionnaire (SF-MPQ) and a Visual Analogue Scale (VAS). Psychosocial burden was evaluated using the Hospital Anxiety and Depression Scale (HADS), the Anxiety Generalized Disorder-7 (GAD-7), the Patient Health Questionnaire-9 (PHQ-9), the Dermatology Life Quality Index (DLQI) and the 6-Item Stigmatization Scale (6-ISS). **Results:** Individuals with AD who reported cutaneous pain in the past week scored significantly higher in HADS (*p* < 0.001), HADS-A (*p* < 0.001), HADS-D (*p* = 0.002), GAD-7 (*p* < 0.001), PHQ-9 (*p* < 0.001), DLQI (*p* < 0.001) and 6-ISS (*p* < 0.001) than the rest of the cohort. More individuals with cutaneous pain had anxiety (36 (48.0%) vs. 7 (18.4%), *p* = 0.002), depression (21 (28.0%) vs. 2 (5.3%), *p* = 0.006) and abnormal HADS scores (46 (61.3%) vs. 9 (23.7%), *p* < 0.001) compared to the rest of participants. Significant correlations were observed between all studied pain assessment tools and all studied psychometric assessments. **Conclusions:** The prevalence and severity of anxiety, depression, stigmatization, and impaired QoL are higher in adults with AD suffering from cutaneous pain compared to those without pain. This symptom is significantly associated with disease burden.

## 1. Introduction

Atopic dermatitis (AD) is one of the most prevalent chronic inflammatory skin diseases affecting 2.6% of the global population [1]. This condition most often begins in infancy, around 3–6 months, but can also persist or appear in adulthood [2]. Recurrent eczematous lesions are found in age-dependent locations and are accompanied not only by profound itch but also, according to recent studies, by cutaneous pain [3,4,5].

Pain is defined as “an unpleasant sensory and emotional experience associated with, or resembling that associated with, actual or potential tissue damage” by the International Association for the Study of Pain (IASP). It commonly manifests as a consequence of a disease or an injury. In numerous conditions, it leads first to acute, then to chronic pain. It is important to distinguish pain as a subjective, burdensome symptom from chronic pain as a separate condition with structural, functional, and chemical alterations in the brain [6,7,8].

The prevalence of cutaneous pain in AD ranges from 42% to 92% according to different reports [3,4,5,9,10]. The mechanism of this symptom remains speculative. It is postulated that this sensation is caused by three abnormal processes: scratching-induced skin damage, peripheral neuron sensitization and elevated levels of inflammatory and excitatory mediators in the skin that activate peripheral neurons [11]. Therefore, cutaneous pain is considered nociceptive (due to tissue damage) and neuropathic (from nerve damage). This sensation correlates with disease severity [4], especially with lesion characteristics including oozing/weeping, cracking and bleeding [12]. In most patients, it is limited to active lesions, particularly in the perioral region and on the hands, corresponding to the densest distribution of nerve endings [4]. Cutaneous pain impairs sleep quality, decreases concentration on assignments, causes irritation, and leads to withdrawal from social events and interactions with others [8,10,13].

AD itself exerts a significant impact on mental health. Adults with AD are at 2- to 3- fold higher risk of developing anxiety and depression compared with the healthy population [14]. Additionally, owing to the visibility of the lesions and the chronic course of the disease, AD patients often feel stigmatized because of their disease [15]. This condition negatively affects quality of life (QoL), with a more pronounced effect as disease severity increases [16]. QoL in AD is lower than in other chronic skin diseases, such as vitiligo and urticaria, and when compared to the healthy controls [16]. Numerous mechanisms are responsible for the detrimental impact of atopic dermatitis on mental health, including sleep difficulties, inflammation, coping strategies and inadequately controlled symptoms [14]. Itch is considered the most burdensome symptom of AD that is associated with psychiatric comorbidities [17,18]. It can negatively influence sleep quality by causing difficulties falling asleep, frequent nighttime awakenings and sleepiness during the day [18]. Sleep disturbance is associated with anxiety and depression symptoms [19]. The majority of studies focus on itch, while the influence of cutaneous pain on mental health remains largely unexplored. Therefore, the objective of this study was to evaluate the impact of cutaneous pain on the prevalence and intensity of clinical symptoms of anxiety, depression, stigmatization, and QoL in adults with AD.

## 2. Materials and Methods

The studied population was recruited at the Department of Dermatology, Venerology, and Allergology of Wroclaw Medical University in Wroclaw, Poland, and the Department of Dermatology at the University Hospital in Krakow, Poland, between March 2023 and January 2024. Methods have already been described in detail in our study on cutaneous pain in psoriasis [20]. Briefly, all patients were asked to complete a designed questionnaire and were examined by a dermatologist. Inclusion criteria involved a diagnosis of AD according to the Hanifin and Rajka criteria [21] and the ability to read and write in Polish. Exclusion criteria included concomitant skin disease causing pain, age below 18 years and cognitive deficit hindering the completion of the questionnaire. The study was conducted in line with guidelines for human studies and the World Medical Association of Helsinki and was approved by the local Ethical Committee of Wroclaw University of Medicine (KB-234/2023).

### 2.1. Disease Severity Assessment

The severity of AD was evaluated using the Eczema Area and Severity Index (EASI) (range 0–72 points), which considers the following characteristics of skin lesions: erythema, induration/papulation, lichenification, excoriation, and the area involved [17]. AD was considered mild when the EASI score was 1.1 to 7 points, moderate when the EASI was 7.1 to 21 points, severe when the EASI was 21.1 to 50 points, and very severe when the EASI was greater than 51 points [22].

### 2.2. Pain and Itch Assessment

Firstly, all participants were asked about the presence of cutaneous pain and itch in the past week. In case of a positive answer, they were requested to specify the intensity of the most severe cutaneous pain and itch using a Numerical Rating Scale (NRS). Additionally, they were asked to complete a Short Form McGill Pain Questionnaire (SF-MPQ) and a Visual Analogue Scale (VAS) for pain assessment.

NRS (range 0–10 points) is a one-dimensional tool, in which 0 represents no pain, while 10 represents the most severe pain imaginable. SF-MPQ (range 0–45 points) consists of 11 sensory descriptors of pain and 4 affective descriptors of pain, each scored from 0 (no pain) to 3 points (severe), providing a multidimensional assessment of pain and is widely used in both research and clinical settings. VAS (range 0–10 points) is a horizontal scale, in which 0 stands for no pain, whereas 10 corresponds to the most intense pain imaginable [23].

### 2.3. Psychometric Assessment

Psychosocial burden of cutaneous pain in AD was evaluated using the Hospital Anxiety and Depression Scale (HADS), the Patient Health Questionnaire-9 (PHQ-9), the Anxiety Generalized Disorder-7 (GAD-7), the Dermatology Life Quality Index (DLQI) and the 6-Item Stigmatization Scale (6-ISS).

HADS (range 0–42 points) consists of two subscales measuring the symptoms of anxiety (HADS Anxiety, HADS-A) and depression (HADS Depression, HADS-D) over the last two weeks. Each consists of 7 questions with responses rated on a 0 to 3-point scale. An abnormal result is a score equal to or greater than 11 points. Diagnosis of anxiety or depression is suggested when the score on the corresponding subscale is 8 points or higher [24].

GAD-7 (range 0–21 points) is a screening tool for generalized anxiety disorder. The instrument includes 7 queries scored from 0 to 3 points, where 0 points stand for “not at all”, 1 point “several days”, 2 points “over half the days”, 3 points “nearly every day”. The results of 5 to 9 points indicate mild anxiety, 10 to 14 points moderate anxiety, and 15 to 21 points severe anxiety [25].

PHQ-9 (range 0–27 points) assesses the prevalence of depression symptoms according to the diagnostic criteria for major depressive disorder. The questionnaire contains 9 questions rated from 0 to 3 points, where 0 indicates “not at all”, 1 point “several days”, 2 points “over half the days”, and 3 points “nearly every day”. The cut-off scores of 5, 10, 15, and 20 points were used to determine depression as mild, moderate, moderately severe and severe. The result of 10 points or higher suggested the presence of major depressive disorder [26].

A Polish version of the DLQI (range 0–30 points) was used to assess the impact of the skin disease on QoL over the past 2 weeks, covering symptoms and feelings, daily activities, recreation, personal relationships, work- and school-related concerns, and treatment side effects. The instrument includes 10 questions, scoring from 0 to 3 points, where 0 corresponds to “not at all”, 1 point “a little”, 2 points “a lot”, and 3 points “very much”. The interpretation of the results is: 0–1 points indicate minimal impact on QoL; 2–5 points, small impact; 6–10 points, moderate impact; 11–20 points, large impact; 21–30 points, extremely large impact [27].

6-ISS (range 0–18 points) is a tool designed to evaluate the following dimensions of stigmatization caused by a dermatological condition: apprehension of exclusion, sensitivity to other people’s attitudes, sentiment of being flawed, negative beliefs, culpability and secretiveness. The instrument involves 6 items, each rated from 0 to 3 points, where 0 points represent “not at all’, 1 point “sometimes”, 2 points “very often”, 3 points “always”. Higher scores suggest greater stigmatization associated with dermatological disease [28].

### 2.4. Statistical Analysis

Firstly, all data were checked for normality using the Shapiro–Wilk test. The minimum, maximum, mean and standard deviation were calculated for each variable. Depending on the normality of the distribution of the data, either the T-Student test or the Mann–Whitney U test was used to compare quantitative variables between two groups. Spearman and Pearson coefficients were used to assess correlations. Qualitative data were analyzed using the Chi-squared test. ANOVA or Kruskal–Wallis test was used to compare differences between more than two groups. Multivariable generalized linear regression models were developed to assess the association between cutaneous pain (measured by NRS) and psychometric variables. All models controlled for the effect of disease severity (EASI), age, sex and the disease duration. In all models, there was no multicollinearity among predictors, as the variance inflation factor was below 3. A *p*-value of ≤0.05 was considered statistically significant. IBM SPSS Statistics version 26 (SPSS Inc., Chicago, IL, USA) was used to conduct all statistical analyses.

### 2.5. Atopic Dermatitis Patients Studied with Demographics and Clinical Data

Finally, 113 AD patients met the inclusion criteria, completed all necessary questionnaires (93.38% response rate) and were included in the analysis. The study group comprised 113 adults (61% female, mean age 34.48 ± 13.20 years). In the entire cohort, the mean EASI score was 13.87 ± 12.90 points with the following distribution: 47 (41.6%) of participants had mild AD, 36 (31.9%) moderate AD, 29 (25.7%) severe AD, and 1 (0.9%) very severe AD. The mean disease duration was 20.02 ± 14.89 years. 41 (36.3%) of participants had a positive family history of AD. In the whole population, 75 (66.4%) individuals reported cutaneous pain in the past week. The mean intensity of cutaneous pain was 3.57 ± 3.14 points on the NRS; 2.95 ± 3.13 points on the VAS; and 9.51 ± 10.96 points on the SF-MPQ, including 7.59 ± 8.49 points on the sensory subscale of SF-MPQ and 1.92 ± 2.82 points on the affective subscale of SF-MPQ. Females scored significantly higher than males on the NRS (4.07 ± 3.18 points vs 2.79 ± 2.94 points, *p* = 0.025), sensory subscale of SF-MPQ (8.91 ± 8.94 points vs. 5.52 ± 7.34 points, *p* = 0.038) and VAS (3.47 ± 3.32 points vs. 2.11 ± 2.63 points, *p* = 0.02). The prevalence and intensity of cutaneous pain, as measured with the remaining pain assessment tools, were also higher in women, but did not reach statistical significance. A total of 105 participants (92.9%) reported itch in the past week with a mean intensity of 5.66 ± 2.86 points on the NRS (Table 1).

## 3. Results

The mean HADS score in the whole group was 12.25 ± 7.89 points, with females obtaining significantly higher scores than males (13.42 ± 7.80 vs. 10.41 ± 7.75, *p* = 0.047). As far as anxiety assessment is concerned, the mean HADS-A score in the studied population was 7.08 ± 4.53 points, with 7.71 ± 4.46 points among females and 6.09 ± 4.52 points among males, while the average GAD-7 score was 6.43 ± 5.56 points, including 6.96 ± 5.60 points in women and 5.61 ± 5.47 points in men. Regarding the evaluation of depression in the entire cohort, the mean HADS-D was 5.18 ± 4.06 points, comprising 5.72 ± 4.14 points in women and 4.32 ± 3.83 points in men, while the average PHQ-9 score was 7.82 ± 6.01 points, involving 8.54 ± 5.66 points in females and 6.70 ± 6.43 points in males. In the QoL assessment, the mean DLQI was 9.49 ± 8.21 points across the whole group, with 10.17 ± 8.13 points in females and 8.41 ± 8.32 points in males. Women scored higher in the evaluation of anxiety, depression and QoL, but without significant differences between genders. They felt significantly more stigmatized by AD than men, as evaluated by 6-ISS (5.01 ± 3.72 points vs. 3.55 ± 3.31 points, *p* = 0.035) (Table 2).

Individuals with AD who reported cutaneous pain in the past week obtained significantly higher HADS scores (14.19 ± 7.85 points vs. 8.42 ± 6.50 points, *p* < 0.001). Moreover, they had more severe symptoms of anxiety estimated by HADS-A (8.25 ± 4.33 points vs. 4.76 ± 4.05 points, *p* < 0.001) and GAD-7 (7.69 ± 5.28 points vs. 3.95 ± 5.32 points, *p* < 0.001). They were also more depressed, as assessed by HADS-D (5.95 ± 4.31 points vs. 3.66 ± 3.05 points, *p* = 0.002) and PHQ-9 (9.77 ± 5.60 points vs. 3.97 ± 4.87 points, *p* < 0.001). Furthermore, they had a more impaired QoL measured by DLQI (12.61 ± 7.91 points vs. 3.32 ± 4.49 points, *p* < 0.001) and more pronounced symptoms of stigmatization on 6-ISS (5.63 ± 3.60 points vs. 2.11 ± 2.30, *p* < 0.001) than the rest of the cohort. AD severity measured by EASI was greater in patients with cutaneous pain (17.40 ± 14.01 points vs 6.90 ± 6.02 points, *p* < 0.001) as well as the intensity of itch (6.67 ± 2.33 points vs 3.68 ± 2.79 points, *p* < 0.001). There were no significant differences in age and sex between the two groups (Table 3). In multivariable regression models, controlled for the effect of disease severity (EASI), age, sex and the disease duration, pain NRS was a predictor of HADS-A (*p* = 0.05), PHQ-9 (*p* = 0.002), DLQI (*p* < 0.001) and 6-ISS (*p* = 0.015).

Of the 75 participants who reported cutaneous pain, 46 (61.3%) had abnormal HADS scores. 36 (48%) had symptoms of clinically probable anxiety determined by HADS-A scores, which was significantly more than in those without pain (7 (18.4%); *p* = 0.002). Moreover, 20 (26.7%) of the AD patients with cutaneous pain had symptoms of minimal anxiety, 33 (44.0%) mild anxiety, 13 (17.3%) moderate anxiety, while 9 (12.0%) had symptoms of severe anxiety, defined by GAD-7 scores, which was significantly more than in the group of subjects without pain (*p* < 0.001). 21 (28%) of individuals suffering from cutaneous pain had symptoms of probable depression, as evaluated by HADS-D, compared to only 2 (5.3%) in the rest of the study cohort (*p* = 0.006). Additionally, 12 (16.0%) of AD patients with pain had symptoms of minimal depression, 26 (34.7%) mild depression, 23 (30.7%) moderate depression, 9 (12.0%) moderately severe and 5 (6.7%) severe depression classified by PHQ-9 scores, which was significantly higher than in the group of participants without pain (*p* < 0.001). Furthermore, 6 (8.0%) of AD patients with cutaneous pain reported minimal impact on QoL, 10 (13.3%) small impact, 15 (20.0%) moderate impact, 26 (34.7%) large impact, 18 (24.0%) extremely large impact on QoL, which was also significantly different from distribution of QoL in subjects without cutaneous pain (*p* < 0.001) (Table 4).

Significant correlations were observed between all studied pain assessment tools (NRS in the past week, SF-MPQ, SF-MPQ sensory subscale, SF-MPQ affective subscale, VAS) and all studied psychometric assessments (HADS, HADS-A, HADS-D, GAD7, PHQ-9, DLQI). The strongest correlation was observed between the sensory component of SF-MPQ and DLQI (*r* = 0.71; *p* < 0.001), while the weakest was between NRS and HADS-A and HADS-D (*r* = 0.23; *p* < 0.05) (Table 5).

## 4. Discussion

In this study, AD patients with cutaneous pain had more severe symptoms of anxiety, depression, stigmatization, and more impaired QoL than participants without pain. Clinically probable depression, anxiety, and impaired QoL were also more prevalent among individuals with painful AD. Additionally, significant associations between the intensity of cutaneous pain and the symptoms of anxiety, depression, stigmatization, and impairment of QoL were found. Our results provide evidence that cutaneous pain might be an important factor in the psychosocial burden of AD.

In our study, 66% of participants reported cutaneous pain in the past week with a mean intensity of 3.57 ± 3.14 points on the NRS, which is consistent with previous reports. For example, in the study by Vakharia et al. [5], the prevalence of cutaneous pain was 42.7%, with a mean intensity of 2.4 points on the NRS. In children, the prevalence of cutaneous pain in the past week was 46.3%, with a median intensity of 5.5 points on the NRS [12]. The highest prevalence of cutaneous pain in AD of 92.2% was reported by Pojawa-Gołąb et al. [10] in an online survey.

The literature about the impact of pain on mental health in AD is limited, but our findings are consistent with the existing reports. For instance, in the study by Vakharia et al. [5] on 305 AD patients, cutaneous pain intensity significantly correlated with DLQI (*r* = 0.45) and PHQ-9 (*r* = 0.36). Similarly, in the survey by Cheng et al. [12] on 240 children with AD, the severity of cutaneous pain reported by parents and children themselves was significantly associated with the Children’s Dermatology Life Quality Index. In addition, cutaneous pain in AD was found to be one of the predictors of persistent depressive symptoms in the study of Chatrath S et al. [29].

The association between AD and psychiatric comorbidities is well-established. For example, the global prevalence of anxiety and depression in individuals with AD is 21% and 17%, respectively, according to a recent meta-analysis [30]. Moreover, adults with this disease are at a 17% higher risk of developing new anxiety and 14% higher risk of developing new depression [31]. Depressive symptoms fluctuate in time with the symptoms of AD involving itch and pain [14,29]. Most patients report exacerbations of mental health diseases during relapses of AD [14]. The combination of itch, anxiety, social isolation, psychological stress, and depression creates a vicious cycle in AD patients [32]. The impact of mental health comorbidities is tremendous as they reduce adherence to treatment and can negatively influence disease management [14].

In our study, a third of AD patients with cutaneous pain had symptoms of clinically probable depression, while 19% reported symptoms of moderately severe and severe depression, and this was significantly more than in individuals without pain. However, although the correlations between pain severity and depressive symptoms were statistically significant, they were not very strong, particularly between NRS and HADS-D. Cutaneous pain is not considered in research on chronic pain conditions [33]. However, if we compare the prevalence of depression in this study to other diseases with chronic pain, it would be similar to musculoskeletal pain (30%) [34], osteoarthritis (29%) [33], spondyloarthritis (29%) [33], but higher than that of patients with rheumatoid arthritis (27%) [33], and psoriasis (21%) [20]. The coexistence of depression and pain can be explained by common pathophysiology involving the same neurotransmitters- serotonin and noradrenaline. They act as modulators of pain stimuli in the periaqueductal area and the limbic system corresponding to “descending inhibition” of pain transmission. Furthermore, antidepressants that regulate the uptake of these neurotransmitters have analgesic properties despite the existence of depression [35].

We demonstrated that nearly half of AD patients with cutaneous pain had symptoms of clinically probable anxiety, while 12% of them reported symptoms of severe anxiety, significantly more than participants without pain. We also found statistically significant correlations between pain and severity of anxiety symptoms; however, they were not very strong, especially between NRS and HADS-A. The prevalence of anxiety in painful AD was comparable to that of chronic pelvic pain (52%) [33], while higher than in arthritis (35%) [36] and in psoriasis patients with cutaneous pain (41%) [20]. The possible explanation of the concurrence of pain and anxiety is that they both serve a survival role, alerting individuals to danger [35]. Moreover, anxiety is responsible for cognitive beliefs of hypervigilance, catastrophizing, and avoidance of fear in chronic pain [35].

In our survey, 58% of AD patients with cutaneous pain reported large and extremely large impairment of QoL, and strong associations between pain intensity and quality of life were found. Silverberg et al. [37] in a cross-sectional, population-based study in the USA, demonstrated that AD, in particular moderate-to-severe AD, was more burdensome than chronic diseases such as hypertension, diabetes, and heart disease. This disease led to social isolation, influenced activity and limited lifestyle. Chronic pain, particularly neuropathic and multisite, was found to interfere with work, mood, sleep, walking activity, general activity, and enjoyment of life [38]. Pain and QoL share common characteristics, including affective, cognitive, behavioural, motivational, and physical components [39], which could explain their coexistence.

The mechanism by which cutaneous pain is associated with psychosocial burden in atopic dermatitis is not known. The plausible explanation is provided by the biopsychosocial model of pain, which involves a dynamic interplay between biological, social, and psychological factors in chronic pain conditions [40]. It is well-established that pain is associated with symptoms of anxiety, depression and sleep problems, but it is less known that these mental health conditions also predispose to pain. Pain causes changes in the peripheral and central nervous systems, including sensitization and the creation of new connections between nerves. Some of these alterations are induced and maintained by psychological influences [40]. Importantly, successful treatment of pain might reverse these changes [34].

Pain can co-occur with itch in up to 78% of AD patients [4]. Itch can lead to sleep disturbance, which is associated with depressive and anxiety symptoms [19]. Many factors may explain insomnia in AD patients, including scratching behaviours, disrupted circadian rhythms by inflammation, the release of cortisol, and inappropriate sleep habits [14].

Additionally, sleep disturbance negatively influences behaviour and mood during the day [41]. In our study, itch intensity was significantly greater in patients with cutaneous pain. Therefore, we need to acknowledge that itch has a potential confounding role in the estimation of the impact of cutaneous pain on mental health burden in AD.

Our study adds to the existing literature that cutaneous pain was evaluated using multiple pain assessment instruments, including SF-MPQ, which allows for a better understanding of various dimensions of pain. Moreover, our analysis included multiple psychiatric assessments, including stigmatization, which was not used before in studies on cutaneous pain in AD. Furthermore, the strength of our study is that the diagnosis of AD was confirmed by a dermatologist. 

The main limitation of our study is that anxiety and depression were evaluated by self-report tools that identify symptoms and probable cases; therefore, in future studies, a diagnosis of anxiety or depression should be confirmed by a psychiatric examination. Moreover, our cohort was ethnically homogeneous, comprised only adults, and was recruited at tertiary care centres, which may have introduced selection bias, and consequently, the results cannot be generalized to all patients with AD, particularly those treated in primary care. The cross-sectional character of our study can be considered a further important limitation. The relationships between cutaneous pain and anxiety, depression, and quality of life are likely to be bidirectional and need to be verified in further longitudinal or interventional studies. We also acknowledge that no correction for multiple comparisons was applied, which increases the risk of type I error. However, given the exploratory and descriptive nature of this cross-sectional study, we treated all analyses as hypothesis-generating rather than confirmatory. Moreover, we acknowledge that findings with *p*-values between 0.05 and 0.01 should be interpreted conservatively, while the main findings with *p* < 0.001 are likely robust.

## 5. Conclusions

To conclude, AD patients with cutaneous pain are more likely to develop anxiety, depression, stigmatization and impaired QoL. Cutaneous pain is associated with greater psychosocial burden. Our results underline that health care providers should ask individuals with AD about cutaneous pain and consistently encourage conversations about the symptoms of psychiatric disorders. They should be trained on how to effectively manage not only symptoms of AD but also associated mental health comorbidities and identify those who would benefit from referrals to mental health specialists. Our study demonstrates that AD is a multifaceted disease that requires a multidisciplinary management. Further investigations are needed to identify the exact mechanisms of cutaneous pain and to optimize its management. Future studies should also clarify if successful treatment of this symptom improves associated psychiatric comorbidities.

## Figures and Tables

**Table 1:** Group characteristics.

Characteristics	Whole Population (*n* = 113)	Females (*n* = 69)	Males (*n* = 44)	*p*
Age, years (mean ± SD)	34.48 ± 13.20	33.77 ± 11.91	35.59 ± 15.07	NS
EASI [points] (mean ± SD)	13.87 ± 12.90	14.45 ± 14.34	12.95 ± 10.34	NS
Disease duration, years (mean ± SD)	20.02 ± 14.89	20.58 ± 15.73	19.14 ± 13.60	NS
Family history of AD *n* (%)	41 (36.3)	25 (37.7)	15 (34.1)	NS
Pain in the past week *n* (%)	75 (66.4)	50 (72.5)	25 (56.8)	NS
Pain NRS in the past week [points] (mean ± SD)	3.57 ± 3.14	4.07 ± 3.18	2.79 ± 2.94	**0.025**
SF-MPQ [points] (mean ± SD)	9.51 ± 10.96	11.12 ± 11.57	7.00 ± 9.53	NS
SF-MPQ sensory [points] (mean ± SD)	7.59 ± 8.49	8.91 ± 8.94	5.52 ± 7.34	**0.038**
SF-MPQ affective [points] (mean ± SD)	1.92 ± 2.82	2.20 ± 3.00	1.48 ± 2.47	NS
VAS [points] (mean ± SD)	2.95 ± 3.13	3.47 ± 3.32	2.11 ± 2.63	**0.021**
Itch in the past week (%)	105 (92.9)	65 (94.2)	40 (90.9)	NS
Itch NRS in the past week [points] (mean ± SD)	5.66 ± 2.86	5.97 ± 2.79	5.18 ± 2.93	0.050

EASI—Eczema Area and Severity Index; NRS—Numerical Rating Scale; SD—standard deviation; SF-MPQ—Short Form McGill Pain Questionnaire; VAS—Visual Analogue Scale; NS—not significant.

**Table 2:** Psychosocial burden of cutaneous pain in the whole AD patients, males and females.

Characteristic (Mean ± SD)	Whole Population (*n* = 113)	Females (*n* = 69)	Males (*n* = 44)	*p*
HADS [points]	12.25 ± 7.89	13.42 ± 7.80	10.41 ± 7.75	**0.047**
HADS-A [points]	7.08 ± 4.53	7.71 ± 4.46	6.09 ± 4.52	NS
HADS-D [points]	5.18 ± 4.06	5.72 ± 4.14	4.32 ± 3.83	NS
GAD-7 [points]	6.43 ± 5.56	6.96 ± 5.60	5.61 ± 5.47	NS
PHQ-9 [points]	7.82 ± 6.01	8.54 ± 5.66	6.70 ± 6.43	NS
DLQI [points]	9.49 ± 8.21	10.17 ± 8.13	8.41 ± 8.32	NS
6-ISS [points]	4.44 ± 3.62	5.01 ± 3.72	3.55 ± 3.31	**0.035**

SD—standard deviation; HADS—Hospital Anxiety and Depression Scale; A—anxiety; D—depression; GAD-7—Generalized Anxiety Disorder-7; PHQ-9—Patient Health Questionnaire-9; DLQI—Dermatology Life Quality Index; 6-ISS—6-Item Stigmatization Scale, NS—not significant.

**Table 3:** Differences between atopic dermatitis patients with and without cutaneous pain.

Characteristic (Mean ± SD)	Cutaneous Pain in the Past Week (*n* = 75)	No Cutaneous Pain in the Past Week (*n* = 38)	*p*
HADS [points]	14.19 ± 7.85	8.42 ± 6.50	<0.001
HADS-A [points]	8.25 ± 4.33	4.76 ± 4.05	<0.001
HADS-D [points]	5.95 ± 4.31	3.66 ± 3.05	0.002
GAD-7 [points]	7.69 ± 5.28	3.95 ± 5.32	<0.001
PHQ-9 [points]	9.77 ± 5.60	3.97 ± 4.87	<0.001
DLQI [points]	12.61 ± 7.91	3.32 ± 4.49	<0.001
6-ISS [points]	5.63 ± 3.60	2.11 ± 2.30	<0.001
EASI [points]	17.40 ± 14.01	6.90 ± 6.02	<0.001
Age [years]	35.87 ± 13.00	31.74 ± 13.32	NS
Sex *n* (%)			
Female	50 (66.7%)	19 (50%)	NS
Male	25 (33.3%)	19 (50%)	
Disease duration [years]	20.65 ± 14.71	18.76 ± 15.37	NS
Itch NRS [points]	6.67 ± 2.33	3.68 ± 2.79	<0.001

SD—standard deviation; HADS—Hospital Anxiety and Depression Scale; A—anxiety; D—depression; GAD-7—Generalized Anxiety Disorder-7; PHQ-9—Patient Health Questionnaire-9; DLQI—Dermatology Life Quality Index; 6-ISS—6-Item Stigmatization Scale; EASI—Eczema Area and Severity Index; NRS—Numerical Rating Scale; NS—not significant.

**Table 4:** Prevalence of anxiety, depression, and impact on the quality of life.

Characteristic (Mean ± SD)	Cutaneous Pain in the Past Week (*n* = 75)	No Cutaneous Pain in the Past Week (*n* = 38)	*p*
HADS abnormal score	46 (61.3%)	9 (23.7%)	**<0.001**
**Anxiety**			
HADS-A	36 (48.0%)	7 (18.4%)	**0.002**
GAD-7			
Minimal anxiety *	20 (26.7%)	28 (73.7%)	**<0.001**
Mild anxiety	33 (44.0%)	4 (10.5%)	
Moderate anxiety	13 (17.3%)	2 (5.3%)	
Severe anxiety	9 (12.0%)	4 (10.5%)	
**Depression**			
HADS-D	21 (28.0%)	2 (5.3%)	**0.006**
PHQ-9			
Minimal depression **	12 (16.0%)	29 (76.3%)	**<0.001**
Mild depression	26 (34.7%)	2 (5.3%)	
Moderate depression	23 (30.7%)	5 (13.2%)	
Moderately severe depression	9 (12.0%)	1 (2.6%)	
Severe depression	5 (6.7%)	1 (2.6%)	
**Quality of life**			
Minimal impact	6 (8.0%)	20 (52.6%)	**<0.001**
Small impact	10 (13.3%)	11 (28.9%)	
Moderate impact	15 (20.0%)	3 (7.9%)	
Large impact	26 (34.7%)	4 (10.5%)	
Extremely large impact	18 (24.0%)	0 (0.0%)	

SD—standard deviation; HADS—Hospital Anxiety and Depression Scale; A—anxiety; D—depression; GAD-7—Generalized Anxiety Disorder-7; PHQ-9—Patient Health Questionnaire-9. * Categories of anxiety were derived from the established GAD-7 cut-off scores: 0 to 4 points minimal anxiety; 5 to 9 points mild anxiety, 10 to 14 points moderate anxiety, and 15 to 21 points severe anxiety. ** Categories of depression were derived from the established PHQ-9 cut-off scores: 0 to 4 points minimal depression; 5 to 9 points mild depression, 10 to 14 points moderate depression; 15 to 19 points moderately severe depression and over 20 points severe depression.

**Table 5:** Correlations between cutaneous pain intensity and psychometric assessments.

Characteristics	Pain NRS in the Past Week	SF-MPQ Total Score	SF-MPQ Sensory	SF-MPQ Affective	VAS
HADS	0.25 **	0.44 ***	0.43 ***	0.40 ***	0.39 ***
HADS-A	0.23 *	0.38 ***	0.39 ***	0.31 ***	0.36 ***
HADS-D	0.23 *	0.44 ***	0.42 ***	0.44 ***	0.36 ***
GAD-7	0.26 **	0.35 ***	0.34 ***	0.31 ***	0.31 **
PHQ-9	0.38 ***	0.53 ***	0.52 ***	0.49 ***	0.46 ***
DLQI	0.67 ***	0.71 ***	0.72 ***	0.60 ***	0.65 ***

HADS—Hospital Anxiety and Depression Scale; A—anxiety; D—depression; GAD-7—Generalized Anxiety Disorder-7; PHQ-9—Patient Health Questionnaire-9; DLQI—Dermatology Life Quality Index; NRS—Numerical Rating Scale; SF-MPQ—Short Form McGill Pain Questionnaire; VAS—Visual Analogue Scale. *** *p* < 0.001; ** *p* < 0.01; * *p* < 0.05.

## Data Availability

The datasets analyzed or generated in the current study are available from the corresponding author upon reasonable request.
